# Relationship of vascular maturation in breast cancer blood vessels to vascular density and metastasis, assessed by expression of a novel basement membrane component, LH39

**DOI:** 10.1054/bjoc.1999.1010

**Published:** 2000-02

**Authors:** S Kakolyris, S B Fox, M Koukourakis, A Giatromanolaki, N Brown, R D Leek, M Taylor, I M Leigh, K C Gatter, A L Harris

**Affiliations:** Department of Cellular Science and ICRF Molecular Oncology Laboratory, John Radcliffe Hospital, University of Oxford, Oxford, OX3 9DU, UK; ICRF Clinical Oncology Unit, Churchill Hospital, Oxford, OX3 7LJ, UK; Experimental Dermatology Laboratory, The Royal London Hospital, London, E1 1BB, UK; Department of Clinical Oncology and Laboratory of Cancer Biology, University Hospital of Heraklion, PO Box 1352, Heraklion 711 10, Crete, Greece; Anatomical Pathology, Canterbury Health, Christchurch Hospital, Private Bag 1470, Christchurch, New Zealand

**Keywords:** LH39, angiogenesis, vascular maturation, breast cancer

## Abstract

Angiogenesis, the formation of new vessels, has been demonstrated to be an indicator of prognosis in breast cancer patients. The extent of differentiation of the tumour vessels may affect access of peripheral white cells and egress or invasion of tumour cells. This has not been assessed in relation to tumour microvessel density or other variables and may be a marker of vascular remodelling. LH39 is a monoclonal antibody recognizing an epitope located at the lamina lucida of mature small veins and capillaries but not in newly formed vessels. To study vascular differentiation in breast tumours, we examined the vascular maturation index (VMI) in 12 normal and 50 breast carcinomas and this was correlated with different clinicopathological variables including angiogenesis. Mature vessels were defined by staining with antibodies to both LH39 and to CD31, using double immunohistochemistry, whereas immature vessels stained only for CD31. VMI was defined as the % fraction of mature vessels (LH39-positive) / total number of vessels (CD31-positive). The VMI was significantly higher in normal (54–68.5%; median 66.5%) than in tumours (0–47%; median 8.8%) (*P* = 0.0005). There was a significant inverse correlation between the tumour VMI and nodal status (Fisher's exact test, *P* = 0.01) and between high VMI and low thymidine phosphorylase (TP) expression (Mann–Whitney *U* -test, *P* = 0.01). No significant association between VMI and tumour size, oestrogen receptor, epidermal growth factor receptor, grade, angiogenesis, patient age, or E-selectin was seen. There was a significant reduction in relapse-free survival (*P* = 0.01) with high angiogenesis. These findings show that the VMI gives new information on the mechanism of tumour angiogenesis independently from microvessel quantitation, there is a wide variation in the differentiation of tumour vasculature but the degree of capillary differentiation is not associated with quantitative angiogenesis. The VMI identifies a subset of patients who have a high chance of regional node involvement. © 2000 Cancer Research Campaign


*British Journal of Cancer* (2000) **82**(4), 844–851
© 2000 Cancer Research Campaign
DOI: 10.1054/ bjoc.1999.1010, available online at http://www.idealibrary.com on IDEAL®

### Relationship of vascular maturation in breast cancer blood vessels to vascular density and metastasis, assessed by expression of a novel basement membrane component, LH39

**S Kakolyris[1,5,*], SB Fox[1,7,*], M Koukourakis[3], A Giatromanolaki[6], N Brown[2], RD Leek[2], M Taylor[3], IM Leigh[4], KC Gatter[1] and AL Harris[2]**

[1]Department of Cellular Science, and [2]ICRF Molecular Oncology Laboratory, John Radcliffe Hospital, University of Oxford, Oxford OX3 9DU, UK; [3]ICRF Clinical Oncology Unit, Churchill Hospital, Oxford OX3 7LJ, UK; [4]Experimental Dermatology Laboratory, The Royal London Hospital, London E1 1BB, UK; [5]Department of Clinical Oncology and [6]Laboratory of Cancer Biology, University Hospital of Heraklion, PO Box 1352, Heraklion 711 10, Crete, Greece; [7]Anatomical Pathology, Canterbury Health, Private Bag 1470, Christchurch Hospital, Christchurch, New Zealand

**Summary** Angiogenesis, the formation of new vessels, has been demonstrated to be an indicator of prognosis in breast cancer patients. The extent of differentiation of the tumour vessels may affect access of peripheral white cells and egress or invasion of tumour cells. This has not been assessed in relation to tumour microvessel density or other variables and may be a marker of vascular remodelling. LH39 is a monoclonal antibody recognizing an epitope located at the lamina lucida of mature small veins and capillaries but not in newly formed vessels. To study vascular differentiation in breast tumours, we examined the vascular maturation index (VMI) in 12 normal and 50 breast carcinomas and this was correlated with different clinicopathological variables including angiogenesis. Mature vessels were defined by staining with antibodies to both LH39 and to CD31, using double immunohistochemistry, whereas immature vessels stained only for CD31. VMI was defined as the % fraction of mature vessels (LH39-positive) / total number of vessels (CD31-positive). The VMI was significantly higher in normal (54–68.5%; median 66.5%) than in tumours (0–47%; median 8.8%) ($P = 0.0005$). There was a significant inverse correlation between the tumour VMI and nodal status (Fisher's exact test, $P = 0.01$) and between high VMI and low thymidine phosphorylase (TP) expression (Mann–Whitney *U*-test, $P = 0.01$). No significant association between VMI and tumour size, oestrogen receptor, epidermal growth factor receptor, grade, angiogenesis, patient age, or E-selectin was seen. There was a significant reduction in relapse-free survival ($P = 0.01$) with high angiogenesis. These findings show that the VMI gives new information on the mechanism of tumour angiogenesis independently from microvessel quantitation, there is a wide variation in the differentiation of tumour vasculature but the degree of capillary differentiation is not associated with quantitative angiogenesis. The VMI identifies a subset of patients who have a high chance of regional node involvement.
© 2000 Cancer Research Campaign

**Keywords:** LH39; angiogenesis; vascular maturation; breast cancer

The formation of new vessels, angiogenesis, is essential for tumour growth and metastasis (Folkman, 1990). Angiogenic process includes retraction of pericytes, protease dissolution of the capillary basement membrane and extracellular matrix, and through modulation of specific adhesion molecules (Bischoff, 1995), endothelial cell (EC) migration. Sprouts are formed which elongate and fuse to form a network of interconnecting loops (Paweletz and Knierim, 1989). Capillary differentiation, including the formation of an irregular basement membrane composed of abnormal ratios of the normal constituents then occurs, before initiation of the blood flow (Blood and Zetter, 1990; Paku and Paweletz, 1991).

LH39 is an antibody to a basement membrane epitope located in the lamina lucida (Almeida et al, 1992*a*). Normal basement membranes are complex, highly compartmentalized structures composed of three major structural zones (lamina lucida, lamina densa and lamina reticularis) (Laurie and Leblond, 1985). Tumour vasculature is known to consist of an irregular basement membrane, composed of variable amounts of fibronectin, laminin and collagen, depending on the maturation status of the capillary (Paku and Paweletz, 1991). Such structural defects of the microvasculature together with the functional effects of various angiogenic factors account for the permeability of the tumour-associated endothelium. LH39 has recently been demonstrated to be present in the basement membranes of small veins and capillaries within the stroma of normal human organs, but was absent in newly formed vessels of several pathological conditions (e.g. ulcers, granulomas) and in cancer (Almeida et al, 1992*a*, 1992*b*). LH39 was also found to be absent in large and medium-sized arteries and veins of normal, inflammatory or neoplastic tissues (Almeida et al, 1992*b*). This is of additional interest, since other basement membrane compounds, like laminin and collagen type IV are known to be expressed in vessels of all sizes.

*Received 26 October 1998*
*Revised 10 September 1999*
*Accepted 16 September 1999*

*Correspondence to:* AL Harris

* Contributed equally to this work

The formation of a new basement membrane usually comes as a late event, following migration and proliferation of ECs within the sprout (Sholley et al, 1984). The establishment of a mature basement membrane has been shown to be accompanied with restricted EC proliferation, something which is mostly associated with the presence of pericytes in the vessel wall (Orlidge and D'Amore, 1987). Hence, although EC proliferation is required for angiogenesis, it is the migration and remodelling of the existing tissue vascular supply which establish the functional vasculature within the tumour (Fox et al, 1993).

To study further the importance of remodelling the existing vessels for tumour angiogenesis, we have examined the vascular maturation index (VMI) of normal and neoplastic breast tissues by measuring the proportion of the total tumour vasculature highlighted by the EC marker CD31 that also express the basement membrane epitope recognized by the mouse monoclonal antibody LH39 (Almeida et al, 1992*a*, 1992*b*). Thus this ratio will give the proportion of vessels in individual tumours that are undergoing remodelling and are still in an immature state. Clearly ECs produced by proliferation will also be incorporated into the remodelled vasculature.

Cell adhesion molecules (CAMs) and their ligands play a major role in metastasis and angiogenesis (Denton et al, 1992; Nelson et al, 1994). The expression of selectins on endothelium at the tumour periphery, where angiogenesis is most active, may play a direct role in tumour angiogenesis. In vitro evidence suggests that selectins are involved in capillary morphogenesis and that ligand binding to selectins of endothelial cells alters their morphology and function (Luscinskas and Lawler, 1994; Bischoff, 1995). Thymidine phosphorylase (TP), an enzyme originally isolated from platelets and also known as platelet-derived endothelial cell growth factor (PDECGF), has been shown to exhibit a chemotactic and mitogenic capacity on endothelial cells in several angiogenic model systems (Haraguchi et al, 1994; Moghaddam et al, 1995; Folkman, 1996). In breast carcinomas up-regulation of TP has been observed (Fox et al, 1996). Therefore, we have also evaluated the expression of E-selectin and TP, and have compared all these measurements to conventional clinicopathological variables and quantitative angiogenesis.

### MATERIALS AND METHODS

### Patients and tumours

Twelve normal breast samples (from reduction mammoplasties) and 50 consecutive primary breast tumours were collected from Cellular Pathology, John Radcliffe Hospital, Oxford, UK. Twelve out of the 50 tumours also included adjacent normal breast tissue. The specimens were snap-frozen in liquid nitrogen and stored at –70°C. Cryostat sections (7-μm thick) were fixed in acetone for 10 min at room temperature, left to dry overnight and either stained immediately or stored at –20°C until required. Formalin-fixed, paraffin-embedded tissue samples from the same breast tumours were also cut in 4-μm thick sections and mounted onto silane-coated slides for CD31 immunohistochemical analysis. The clinicopathological characteristics of patients and tumours are detailed in Table 1. Patients were treated with lumpectomy ($n$ = 35) or simple mastectomy ($n$ = 15); tamoxifen was given to those ≥ 50 years of age or oestrogen receptor (ER)-positive and <50 years old. In patients < 50 years old, adjuvant cyclophosphamide, methotrexate and 5-fluorouracil (CMF) was administered if tumours were node-positive, or ER-negative and/or ≥ 3 cm. Patients ≥ 50 years with ER-negative, node-positive tumours also received CMF. Twelve patients received chemotherapy, 20 tamoxifen and 18 both. Follow-up (median 70 months (range 10–86 months)) was 3-monthly for 18 months, and 6-monthly until 3 years.

### Immunohistochemistry

The primary antibodies used were for PECAM/CD31, JC70A (Dako, UK); E-selectin/CD62, E1.2 B6 (ascites dil.1/500) from D Haskard, London, UK; lamina lucida antigen, LH39 (supernatant) from I Leigh, London, UK; TP/PG44c (supernatant) from KC Gatter, Oxford, UK. For double-labelling, a three-step, indirect avidin–biotin peroxidase complex (ABC) technique was first performed using LH39, before an alkaline phosphatase anti-alkaline phosphatase (APAAP) staining procedure with JC70 was performed as described below. The specificity of all staining was confirmed by replacing the primary antibodies with an irrelevant isotype antibody.

### LH39/CD31

Seven-micrometre cryostat sections were incubated with LH39 overnight in a moist chamber at 40°C. Secondary antibody, biotinylated goat anti-mouse immunoglobulin (Dako; K492) was applied for 20 min, followed by incubation with streptavidin

**Table 1** Clinicopathological characteristics of patients and tumours

| | |
|---|---|
| Age, years | |
| Median (range) | 55 (32–79) |
| < 50 | 16 |
| ≥ 50 | 34 |
| Surgical treatment | |
| Lumpectomy | 35 |
| Simple mastectomy | 15 |
| Adjuvant treatment | |
| Chemotherapy | 12 |
| Tamoxifen | 20 |
| Both | 18 |
| Lymph nodes, neg/pos | 31/19 |
| Tumour size, cm | |
| Median (range) | 2.4 (0.3–5.5) |
| ≤ 2 | 12 |
| > 2 | 37 |
| Histology | |
| Ductal | 35 |
| Lobular | 4 |
| Others | 11 |
| Grade (ductal) | |
| I | 3 |
| II | 11 |
| III | 21 |
| ER fmol $mg^{-1}$ | |
| Median (range) | 12 (0–314) |
| < 10 | 23 |
| ≥ 10 | 27 |
| EGFR fmol $mg^{-1}$ | |
| Median (range) | 9.2 (0–710) |
| < 20 | 36 |
| ≥ 20 | 14 |
| Survival follow-up, months | |
| Median (range) | 70 (10–86) |
| Deaths/Recurrences | 12/17 |

biotinylated horseradish peroxidase for another 20 min. Peroxidase reaction was developed using diaminobenzidine tetrahydrochloride (DAB; Sigma). Slides were then incubated with JC70 (CD31) for 1 h at room temperature before sequential application of anti-mouse immunoglobulin (Dako; Z259, at 1/50 dilution) and APAAP complex for 30 min each. This step was repeated twice (second and third incubations) for a further 10 min each to enhance the intensity of the final staining. The APAAP reaction was visualized using new fuchsin (Dako; K596) and slides were weakly counterstained with haematoxylin and mounted in aqueous medium. Three 5-min washings between incubations in Tris-buffered saline (TBS) pH 7.2 was performed.

### E-selectin

Immunohistochemistry for E-selectin was performed on the same cryostat sections (multiwell slides each including four serial sections were used), stained with 1.2B6, using the streptavidin–biotin horse-radish peroxidase–DAB technique (Fox et al, 1995*a*). A semi-quantitative analysis was used for assessment of E-selectin expression, as previously described (Fox et al, 1995*a*).

### TP

A standard peroxidase ABC method using DAB as chromogen, was performed to visualize TP staining, with PG44c antibody as primary, as previously described (Fox et al, 1995*b*). Assessment of TP expression was based on both the intensity and extent of staining of either: (a) the tumour cells, or (b) cancer cells and stroma cells (overall TP). Cases were considered TP-positive, if more than 25% of cells displayed moderate staining, as this cut-point has previously been found to be significant in predicting survival in node-positive breast tumours treated with CMF, with high TP expressors showing improved survival (Fox et al, 1996).

### ER and EGFR

These were determined by enzyme-linked immunosorbent assay (ELISA) technique (Abbott Laboratories, USA) and ligand binding of $^{125}$I-EGF respectively. Tumours were considered positive when ER ≥ 10 fmol mg$^{-1}$ cytosolic and EGFR ≥ 20 membrane protein respectively (Fox et al, 1994).

### Quantitation of tumour angiogenesis

A 25-point Chalkley eypiece graticule (Chalkley, 1943) was used to count vascular hot spots identified by scanning the tumour at ×40–100 by two observers over a conference microscope. Microvessels were defined as any immunoreactive EC(s) separate from adjacent microvessels. Vessels within the sclerotic body of the tumour were not included (Weidner et al, 1991). Counting at ×250 magnification (0.155 mm$^2$) was then performed by rotating the graticule in the eyepiece to where the maximum number of dots overlay stained vessels. The mean of the three counts was used in the subsequent analysis and tumours with counts > 7 were considered high vascularity. Tumours with counts of less than 7 were considered as low (0–4) and median (5–6) vascularity. However, low and median vascularity were included in the same group, since previous studies have shown the validity of this categorization (Fox et al, 1995*c*). Chalkley counts were determined without knowledge of patient outcome (Fox et al, 1995*c*).

### VMI

All vessels were stained with anti-CD31 antibody but only a subset stained for LH39. The median number of LH39-positive vessels per field, and the number of LH39-positive vessels in the ‘hot spot’ area (as indicators of maturation), were counted and used for statistical analysis. VMI was defined as the percentage of LH39-stained to CD31-stained vessels, both quantified in the same double-stained section. The index was derived by counting the total number of double (LH39+CD31) and single (CD31)-stained vessels in each tumour section. Thus, the index was LH39-positive vessels/total CD31-positive vessels × 100 to express as a percentage. Counting was done throughout the section (at ×250 magnification), with the Chalkley graticule maintained in one position (the average not the maximal number of vessels in the tissue section was counted). Vessel counting (the criteria for which are described above) was determined independently by two observers. The counts were split by tertiles and the upper third was used as the cut-off for categorical and survival analyses (Fox et al, 1995*c*, 1995*d*).

### Statistical analysis

These were performed using Stata Release 3.1 (Stata Corporation, TX, USA). Fisher’s exact test was used to examine the relationship between categorical variables, and dependent logistic regression with backward analysis for interaction between variables. A non-parametric Mann–Whitney *U*-test was used to compare relationship between TP expression (overall or tumour TP) as categorical variable, and VMI as a continuous tumour variable. Paired or unpaired ranked sums were used for paired or unpaired data respectively. The log-rank test was used to perform univariate survival analyses.

### RESULTS

Positive labelling for LH39 was detected in all normal and neoplastic tissues examined. The staining was evident in the basement membranes of small vessels and epithelial basement membranes. Details of the staining pattern are described below.

### LH39 expression in normal breast (reduction mammoplasty vs tissue adjacent to tumour)

In the 12 examined cases from reduction mammoplasties, LH39 labelled the majority of endothelial basement membranes of small veins and capillaries. The distribution and the intensity of staining for LH39 in normal breast microvasculature was generally homogenous. The VMI in normal breast ranged from 54 to 68.5% (median 66.5%). The staining of vessels with antibodies to both CD31 and LH39 antigen is shown in Figure 1 A and B.

In the 12 cases of normal breast tissue close to invasive tumour, the median VMI was lower than in reduction mammoplasties (52.5%, range 18–75%), and this difference was significant ($P$ = 0.007), unpaired ranked sums, Figure 2, C vs D). Basement membranes surrounding breast ducts and lobules were also strongly positive for LH39.

### LH39 expression in breast carcinomas

In the 50 breast carcinomas examined, VMI was assessed either in the three ‘hot spot’ areas (previously selected for angiogenesis

 © 2000 Cancer Research Campaign

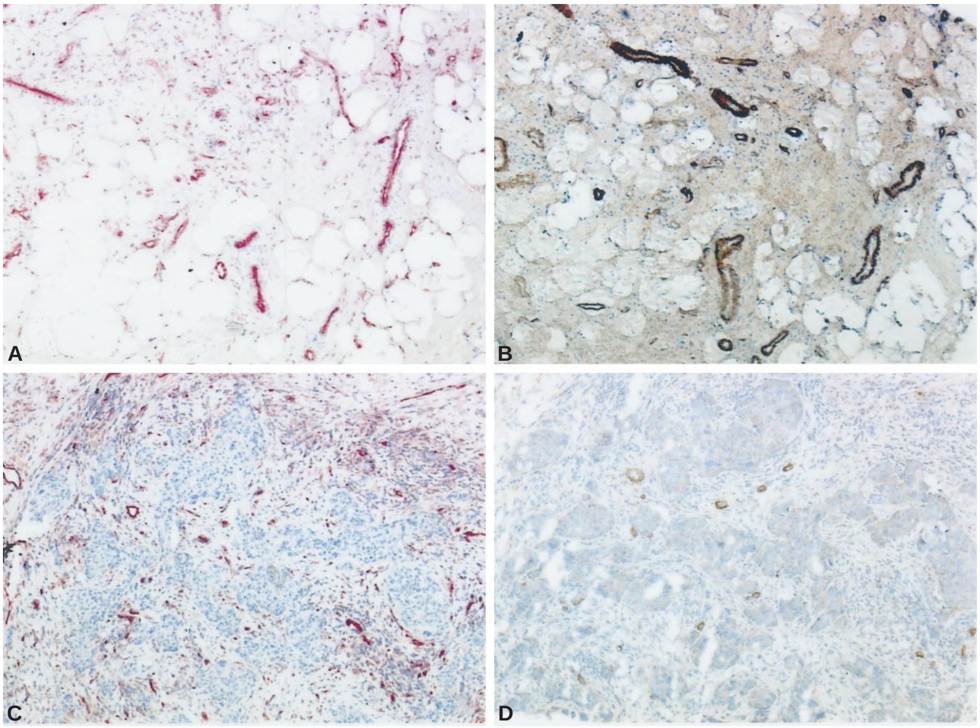

**Figure 1** Single immunohistochemical staining with either CD31 (APAAP) or LH39 (ABC). Normal breast (**A** and **B**) stained with CD31 (red) and LH39 (brown) showing most vessels to be stained with occasional vessels being only CD31-positive. Invasive breast carcinomas stained for and CD31 (red, **C**) and LH39 (brown, **D**) showing most vessels are CD31 single-stained

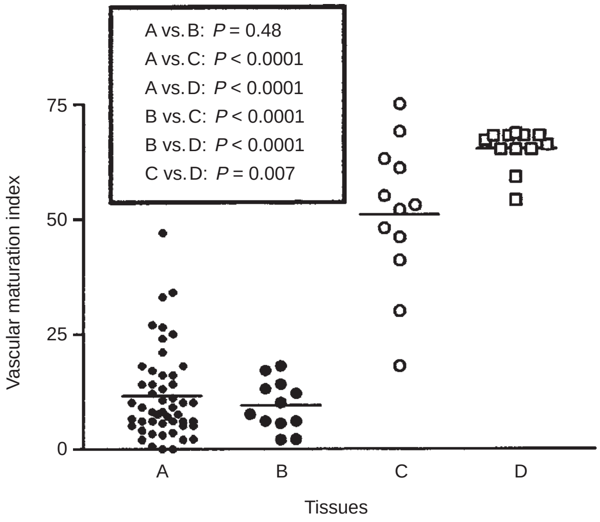

**Figure 2** Scattergram of vascular maturation index (VMI) in all 50 cancer cases (A), in 12 cancer cases with adjacent normal breast tissue (B), in 12 cases with normal breast adjacent to tumour (C), in 12 cases with normal reduction mammoplasty tissue (D). The index represents the ratio of mature vessels expressing LH39 antigen over total vessels detected by CD31 expression assessment) and throughout the tumour. The mean hot spot VMI was 6.1 ± 8, which was lower than the VMI for the whole tumour area, mean 11.4 ± 9, $P$ = 0.0001. Linear regression analysis showed that the hot spot MRI correlated with overall VMI ($r$ = 0.061, $P$ = 0.0001). The latter was considered as more representative in defining vessel maturation in the whole tumour and was subsequently used in the statistical analysis. However, the same analyses for correlation with pathological variables and outcome were analysed using hot spot VMI and none were significant.

Whole area VMI in breast carcinomas ranged from 0 to 47% (median 8.8%), which was significantly lower than that observed in the 12 normal breast cases (range 54–68.5%; median 66.5%, $P$ < 0.0005, unpaired ranked sums) and the 12 cases of normal breast besides tumour (range 18–75%; median 52.5%, $P$ < 0.0005, paired ranked sums). The staining for tumour vessels with antibodies to CD31 and LH39 antigen is shown in Figure 1, C and D. An analysis was performed comparing VMI between all 50 cancer cases (Figure 1A), the 12 cancer cases with adjacent normal breast (Figure 1B), the same cases with normal breast adjacent to invasive tumour (Figure 1C) and 12 normal breast cases taken from reduction mammoplasties (Figure 1D). The difference in VMI between tumour and reduction mammoplasty breast was again highly significant (Figure 1A or B vs C, $P$ < 0.0001, unpaired ranked sums, Figure 2), as was the difference in the tumour and paired adjacent normal tissue (B vs C, $P$ < 0.0001, Figure 2). LH39-positive vessels were identified throughout the tumour body in all examined cases. However, focal differences in the distribution of LH39-positive vessels were observed in three cases at the invasive tumour edge. In those cases, the concentration of LH39-positive vessels at the invasive edge, was higher than that observed in the remaining tumour area and appeared to represent entrapped residual normal tissue. LH39 expression was evident in all tumour epithelial basement membranes, in a similar pattern to that observed in normal breast. Only in some cases with extensive disruption of the basement membranes was focal or occasional LH39 expression seen.

### Expression of E-selectin on breast tumour endothelium

A significant proportion of the tumour endothelial cells also showed positive staining for E-selectin. Hence, 14/50 cases were negative, 21/50 were positive and 15/50 cases presented strong positivity for E-selectin. Expression of E-selectin was not restricted to a particular vessel type but was observed in the endothelial cells of arterioles, venules and small capillaries. However, in 14/36 cases showing immunoreactivity with E-selectin the staining was more prominent at the tumour periphery. In three cases a weak labelling for E-selectin was observed within the neoplastic element of the tumour.

### TP expression

The usual pattern of immunoreactivity for TP/PD-ECGF was both nuclear and cytoplasmic but occasionally only one of these was present. Immunoreactivity was heterogenous, occasionally focal, and often up-regulated at the infiltrating tumour edge. Immunostaining was frequent in the stroma, tumour-associated macrophages and endothelial cells. Tumour cells were positive for TP/PD-ECGF in 24/37 cases, while overall TP/PD-ECGF positivity was detected in 22/37 examined cases with breast carcinomas.

### Relationship of VMI expression to other tumour variables and survival analysis

VMI expression was analysed either as a continuous or as a categorical variable. On splitting VMI into thirds (cut-off points 6% and 13% LH39-positive vessels) we categorized VMI in breast carcinomas as high, medium or low. The Fisher exact tests performed to see the relationship of VMI to different tumour variables revealed a relationship of high VMI to negative lymph node status ($P = 0.01$), but not to other prognostic variables (Table 2). Dependent logistic regression confirmed the only significant factor relating to node involvement was VMI ($P = 0.007$, Table 3). Also stratifying by tertiles 11/19 low VMI (< 6%) were node-posi-

**Table 2** Contingency tables comparing VMI with the clinicopathological characteristics of patients

| | VMI | | |
|---|---|---|---|
| | Low | High | *P*-value |
| Age (years) | | | |
| < 50 | 10 | 6 | |
| ≤ 50 | 24 | 10 | 0.75 |
| Lymph nodes | | | |
| Negative | 17 | 14 | |
| Positive | 17 | 2 | 0.01 |
| Grade | | | |
| I/II | 8 | 5 | |
| III | 14 | 4 | 0.43 |
| Tumour size | | | |
| ≤ 2 cm | 6 | 6 | |
| > 2 cm | 27 | 10 | 0.17 |
| E-selectin | | | |
| Negative | 23 | 13 | |
| Positive | 11 | 3 | 0.5 |
| Angiogenesis | | | |
| ≤ 7 | 26 | 12 | |
| > 7 | 7 | 4 | 0.75 |
| ER[a] | | | |
| < 10 | 14 | 9 | |
| ≤ 10 | 20 | 7 | 0.37 |
| TP (tumour) | | | |
| Negative | 7 | 6 | |
| Positive | 12 | 12 | 0.09 |
| TP (overall) | | | |
| Negative | 3 | 12 | |
| Positive | 12 | 10 | 0.01 |
| EGFR[a] | | | |
| < 20 | 25 | 10 | |
| ≥ 20 | 9 | 6 | 0.52 |

[a]fmol mg$^{-1}$ protein.

**Table 3** Dependent logistic regression on 50 patients examining relationship between positive lymph node status and other clinicopathological variables

| Variable | Odds ratio | 95% CI | *P*-value |
|---|---|---|---|
| Angiogenesis > 7; ≤ 7 | 1.13 | (0.2–5.9) | 0.9 |
| ER[a] ≥ 10; < 10 | 1.6 | (0.3–7.6) | 0.56 |
| EGFR[a] ≥ 20; < 20 | 0.9 | (0.16–5.1) | 0.9 |
| Age ≥ 50; < 50 | 0.33 | (0.1–2.2) | 0.26 |
| TP (overall) positive; negative | 0.8 | (0.15–3.2) | 0.8 |
| Size > 2 cm; ≤ 2 | 3.2 | (0.5–19.6) | 0.2 |
| VMI high; medium; low | 0.2 | (0.1–0.6) | 0.007 |

CI, confidence intervals. [a]fmol mg$^{-1}$ protein.

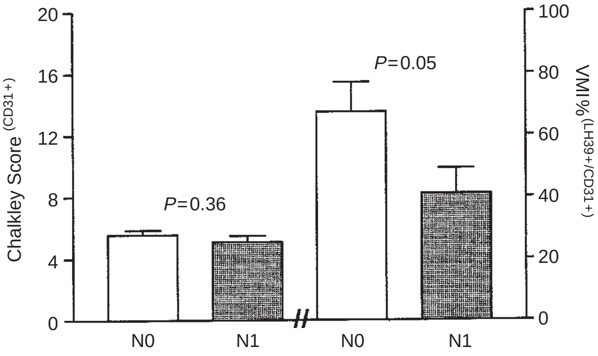

**Figure 3** Correlation of vascular maturation index (VMI) and angiogenesis (as detected by Chalkley score, CS) with nodal (N) involvement. High VMI correlates significantly with negative lymph node status (N0)

*© 2000 Cancer Research Campaign*

tive, 6/15 medium VMI (< 13%) were node-positive, and only 2/16 high VMI (> 13%) were node-positive ($P$ = 0.02, Fisher's exact test). Continuous variable analysis also confirmed that VMI significantly correlated with node involvement. Cases with N0 had a VMI of 13.5 + 10.6, whilst cases with N1 had a VMI of 8.5 + 6.9 (Figure 3, $P$ = 0.05).

The Mann–Whitney $U$-test revealed a significant correlation between high VMI (analysed as continuous variable) and low (negative) overall TP expression (as categorical variable, $P$ = 0.01, Table 2), but not for tumour cell TP expression.

The univariate analysis performed to see significance of each tumour variable in predicting relapse, revealed significance only for angiogenesis ($P$ = 0.01) but not for any VMI category. Similarly, only angiogenesis and lymph node status were found to have significance in predicting survival ($P$ = 0.02 and 0.002 respectively). Variables combining angiogenesis with VMI categories were also produced, and VMI was found to have no impact in defining subgroups with different prognosis in low or high angiogenesis cases. Cases with low angiogenesis had a statistically better prognosis compared to cases with high angiogenesis no matter what the VMI was.

### DISCUSSION

Using a double immunohistochemical labelling technique for EC and basement membrane markers, using a basement membrane component absent from vessels actively involved in neovascularization (Almeida et al, 1992*a*, 1992*b*), we have demonstrated that the degree of vascular maturation in normal and neoplastic breast tissue varies widely.

Normal breast tissues, like other mature normal tissues, have a fully developed vessel network and a quiescent endothelial cell phenotype (Fox et al, 1993; Vartanian and Weidner, 1994). However, the proportion of poorly differentiated vessels observed in the normal breast tissues in this study, which consist largely of reduction mammoplasties, suggests that angiogenesis is occurring to a greater extent in physiologically normal adult tissues, than indicated by endothelial labelling. Thus, although EC proliferation is not characteristic of angiogenesis in normal breast, an angiogenic phenotype defined by the presence of LH39-negative (immature) vesels, indicating a remodelling process, might still occur in normal breast. In the case of breast tissue, the process may be part of the more general tissue alterations, which occur during cyclical hormonal release. Indeed there is increasing evidence that oestrogens effect several steps of the angiogenic process (Morales et al, 1995; Schnaper et al, 1996), including mediation of cytokine-induced adhesion molecule expression in endothelial cells (Caulin-Glazer et al, 1996), molecules important in the remodelling process.

We have suggested previously, based on a relatively low EC proliferation index in breast carcinomas (mean 2.2%), that EC remodelling might be an important mechanism by which human tumours establish a blood supply (Fox et al, 1993). The findings in the present study show a high proportion of poorly differentiated vessels throughout the tumour as well as at the growing edge where dividing EC are present (Fox et al, 1993). Thus, VMI may represent the aggregate of new vessels produced by both division and remodelling. The VMI in the microvessel hot spots was even lower than in the rest of the tumour, reflecting the remodelling of dividing ECs and confirming these areas as the most angiogenic. There was a significant correlation between the hot spot VMI and total VMI suggesting that either the rest of the vasculature developed from the hot spots or that there may be separate vascular remodelling factors acting throughout the tumour in addition to those regulating proliferation at the hot spots. The remodelling of tumour vasculature continues after its formation (Folkman, 1997), reflecting a potential stratagem by which a tumour might match its blood supply to its growing demands. Furthermore, the increase in VMI between tumour, normal near tumour and normal breast is in accordance with the notion that angiogenesis is tightly suppressed in normal tissues but might be stimulated by genetic changes in apparent normal tissues (Deng et al, 1996). Alternatively, this may be an effect of soluble mediators acting in a paracrine mechanism.

Although no significant association between VMI and quantitative angiogenesis, either in the area assessed for vessel differentiation or in the remainder of the tumour (hot spot method) was observed, the VMI measures a different aspect of angiogenesis to vessel number, since it gives the proportion of tumour vessels being remodelled. This is supported by the observed significant inverse association between a low VMI (high vascular remodelling) and positive lymph node status. The association of an immature, poorly differentiated vasculature may also be representative of the lymphatic vessels at the periphery of the tumour. This would facilitate regional spread. Also, the immature vessels themselves may contribute via proteases to local invasion of tumour. The association with nodal status is also in accordance with the possibility that leaky immature vessels are more likely to facilitate tumour cell escape into adjacent lymphatics, a process that would be retarded in more mature vessels.

Although several earlier studies (Bosari et al, 1992; Weidner et al, 1992) found an association of high microvessel density with lymph node involvement, a similar number of studies has found no association, as reported here (Kato et al, 1999; Toi et al, 1994; Van Hoef et al, 1993). The reasons are unclear but probably reflect that there is not a direct relation of the route of lymph node spread to the vasculature, although both processes reflect an aggressive phenotype. There is substantial heterogeneity in breast cancer so that either meta-analysis or much larger studies are needed to resolve this.

High VMI was found to associate with low TP expression. TP, although not a classic growth factor, has been shown to be both chemotactic and mitogenic for endothelial cells and angiogenic in several model systems (Haraguchi et al, 1994; Moghaddam et al, 1995; Folkman, 1996). Therefore, low expression of a mitogenic/angiogenic factor, such as TP, may indicate a less aggressive tumour phenotype. This would possibly facilitate the establishment of a well differentiated/mature tumour vasculature, which by turn associates with good prognostic features, such as regional lymph node negativity. No significant association between VMI and other clinicopathological variables was observed. E-selectin expression was more prominent at the tumour periphery, an area where angiogenesis is most active (Fox et al, 1993), consistent with a reported role in capillary morphogenesis (Nguyen et al, 1993; Kaplanski et al, 1994; Luscinskas and Lawler, 1994; Bischoff, 1995). However, the pathways in this study do not appear to be simultaneously expressed.

A tumour is considered to have 'switched' to an angiogenic phenotype when it alters the balance of angiogenic stimulators and inhibitors, thereby gaining the capacity to establish a blood supply. Tumour angiogenesis is not an ordered process and individual

*© 2000 Cancer Research Campaign*

tumours vary in their ability to form structurally normal vessels (Warren, 1979; Lin et al, 1984; Skinner et al, 1995). Thus, the number of tumour vessels alone will only give one aspect of a tumour's ability to complete the complex angiogenic programme. Although low VMI was associated with regional node spread, tumours that have acquired the ability to form mature vessels, i.e. have a high VMI similar to normal tissues, might be those tumours with a more effective blood supply. Thus, VMI might not only give an index of the proportion of vessels produced by cell division and remodelling (angiogenic index), but might also give an indication as to the function of such vessels. Indeed, particular vascular patterns in lung carcinomas (Pezella et al, 1996), and ocular melanomas (Folberg et al, 1993), which similarly might reflect different qualitative angiogenic phenotypes, have been shown to be associated with differences in prognosis.

Currently, tumour angiogenesis is measured by estimating the number of immunohistochemically identified vessels in angiogenic hot spots (Fox, 1997), however, not all studies have confirmed the utility of this technique. Furthermore, the efficiency of the tumour vasculature will be determined not only by the number but by the quality of the vessels generated. These limitations have encouraged the evaluation of other indirect measures of tumour angiogenesis, such as serum growth factor levels from cancer-bearing patients (Nguyen et al, 1994). The concept of an angiogenic profile encompassing several angiogenic pathways might be more useful and measures of capillary maturation should be considered as a potential part. This might become more important with the advent of anti-angiogenic agents directed against particular processes involved in vascular remodelling, such as EC migration, cell–matrix interactions and secretion of basement membrane (Ingber and Folkman, 1988; Maragoudakis et al, 1988; Nicosia and Bonano, 1991; Sakamoto et al, 1991; Kibbey et al, 1992; Grant et al, 1994).

These observations are also relevant to the efficacy of vascular targeting (Nicosia and Bonano, 1991). This type of therapy, which aims to accutely destroy tumour vasculature with a different phenotype to normal tissues, may be even more effective than expected if such a high proportion of the vasculature is in an immature state. Recent demonstration of tumour regression with growth inhibitory peptides specific for vascular endothelium was hard to explain if only proliferating endothelial cells were inhibited (Sakamoto et al, 1991). However, if over 80% of the vessels are poorly differentiated, as a result of remodelling and endothelial growth, inhibition of these pathways will be more effective than previously expected.

In summary, this study has demonstrated a method for assessing maturation of tumour-associated vessels that are poorly differentiated and may therefore be involved in vascular remodelling. We have shown that this remodelling occurs in normal premenopausal breast but is significantly up-regulated in tumours. We also suggest that measures of capillary maturation might be complementary to microvessel number to aid the identification of patients who might benefit from specific anti-angiogenic therapies or vascular targeting treatment.

### REFERENCES

Almeida BM, Challacombe SJ, Eveson JW, Smith CG and Leigh IM (1992*a*) A novel lamina lucida component of epithelial and endothelial basement membranes detected by LH39 monoclonal antibody. *J Pathol* **166**: 243–253

Almeida BM, Challacombe SJ, Eveson JW, Morgan PR, Purkis PE and Leigh IM (1992*b*) The distribution of LH39 basement membrane epitope in the tumour stroma of oral squamous cell carcinomas. *J Pathol* **166**: 369–374

Bosari S, Lee AKC, DeLellis RA, Wiley BD, Heatley GJ and Silverman ML (1992) Microvessel quantitation and prognosis in invasive breast carcinoma. *Hum Pathol* **23**: 755–761

Bischoff J (1995) Approaches to studying cell adhesion molecules in angiogenesis. *Trends Cell Biol* **5**: 69–74

Blood CH and Zetter BR (1990) Tumor interactions with the vasculature: angiogenesis and tumor metastasis. *Biochim Biophys Acta* **1032**: 89–118

Caulin-Glazer T, Watson C, Pardi R and Bender J (1996) Efects of 17β-estradiol on cytokine induced endothelial cell adhesion molecule expression. *J Clin Invest* **98**: 26–42

Chalkley H (1943) Method for the quantative morphological analysis of tissues. *J Natl Cancer Inst* **4**: 47–53

Deng G, Lu Y, Zlotnikof G, Thor A and Hs S (1996) Loss of heterozygosity in normal tissue adjacent to breast carcinomas. *Science* **274**: 2057–2059

Denton KJ, Stretch JR, Gatter KC and Harris AL (1992) A study of adhesion molecules as markers of progression in malignant melanoma. *J Pathol* **167**: 187–191

Folberg R, Rummelt V, Ginderdeuren R-V, Hwang T, Woolson R, Pe'er J and Gruman L (1993) The prognostic value of tumor blood vessel morphology in primary uveal melanoma. *Ophthalmology* **100**: 1389–1398

Folkman J (1990) What is the evidence that tumours are angiogenesis dependent? *J Natl Cancer Inst* **82**: 4–6

Folkman J (1996) What is the role of thymidine phosphorylase?

Folkman J (1997) New perspectives in clinical oncology from angiogenesis research. *Eur J Cancer* **32A**: 2534–2539

Fox SB (1997) Tumor angiogenesis and prognosis. *Histopathology* **30**: 294–301

Fox SB, Gatter KC, Bicknell R, Going J, Stanton P, Cooke T and Harris AL (1993) Relationship of endothelial cell proliferation to tumor vascularity in human breast cancer. *Cancer Res* **53**: 9161–9163

Fox SB, Smith K, Hollyer J, Greenall M, Hastrich D and Harris AL (1994) The epidermal growth factor receptor as a prognostic marker: results of 370 patients and review of 3009 patients. *Breast Cancer Res Treat* **29**: 41–49

Fox SB, Turner GDH, Gatter KC and Harris AL (1995*a*) The increased expression of adhesion molecules ICAM-3, E and P selectin on breast endothelium. *J Pathol* **177**: 369–376

Fox SB, Moghaddam A, Westwood M, Turley H, Bicknell R, Gatter KC and Harris AL (1995*b*) Platelet-derived endothelial cell growth factor/thymidine phosphorylase expression in normal tissues: an immunohistochemical study. *J Pathol* **176**: 183–190

Fox SB, Leek RD, Weekes MP, Whitehouse RM, Gatter KC, Harris AL (1995*c*) Quantitation and prognostic value of breast cancer angiogenesis: comparison of microvessel density, Chalkley count and computer image analysis. *J Pathol* **177**: 275–283

Fox SB, Turner GDH, Leek RD, Whitehouse RM, Gatter KC and Harris AL (1995*d*) The prognostic value of quantitative angiogenesis in breast cancer and role of adhesion molecule expression in tumour endothelium. *Breast Cancer Res Treat* **36**: 219–226

Fox SB, Westwood M, Moghaddam A, Comley M, Turley H, Whitehouse RM, Bicknell R, Gatter KC and Harris AL (1996) The angiogenic factor platelet-derived endothelial cell growth factor/thymidine phosphorylase is up-regulated in breast cancer epithelium and endothelium. *Br J Cancer* **73**: 275–280

Grant DS, Kibbey MC, Kinsella JL, Cid MC and Kleinman HK (1994) The role of basement membrane in angiogenesis and tumor growth. *Pathol Res Pract* **190**: 854–863

Haraguchi M, Kazutaka M, Uemura K, Sumizawa T, Furukawa, Yamada K and Akiyama S-I (1994) Angiogenic activity of enzymes. *Nature* **368**: 198–200

Ingber D and Folkman J (1988) Inhibition of angiogenesis through modulation of collagen metabolism. *Lab Invest* **59**: 44–51

Kaplanski G, Farnarier C, Benoliel A, Foa C, Kaplanski S and Bongrand P (1994) A novel role for E- and P-selectins: shape of endothelial cell monolayers. *J Cell Sci* **107**: 2449–2457

Kato T, Kimura T, Ishii N, Fujii A, Yamamoto K, Kameoka S, Nishikawa T and Kasajima T (1999) The methodology of quantitation of microvessel density and prognostic value of neovascularization associated with long-term survival in Japanese patients with breast cancer. *Breast Cancer Res Treat* **53**: 19–31

Kibbey MC, Grant DS and Kleinman HK (1992) Role of the SIKVAV site of laminin in promotion of angiogenesis and tumor growth: an in vivo Matrigel model. *J Natl Cancer Inst* **84**: 1633–1638

Laurie GW and Leblond CP (1985) Basement membrane nomenclature. *Nature* **313**: 272–274

© 2000 Cancer Research Campaign

Lin G, Lunderquist A, Hagerstrand I and Boijsen E (1984) Postmortem examination of the blood supply and vascular pattern of small liver metastases in man. *Surgery* **96**: 517–526

Luscinskas FN and Lawler J (1994) Integrins as dynamic regulators of vascular function. *FASEB J* **8**: 929–938

Maragoudakis ME, Sarmonika M and Panoutsakopoulou M (1988) Inhibition of basement membrane biosynthesis prevents angiogenesis. *J Pharmacol Exp Ther* **244**: 729–733

Moghaddam A, Zhang H-T, Fan T-P, Hu D-E, Lees V, Turley H, Fox SB, Gatter KC, Harris AL and Bicknell R (1995) Thymidine phosphorylase is angiogenic and promotes tumour growth. *Proc Natl Acad Sci USA* **92**: 988–1002

Morales DE, McGowan KA, Grant DS, Maheshwari S, Bhartiya D, Cid MC, Kleinman HK and Schnaper HW (1995) Estrogen promotes angiogenic activity in human umbilical vein endothelial cells in vitro and in a murine model. *Circulation* **91**: 755–763

Nelson H, Ramsey P, Donohue J and Wold L (1994) Cell adhesion molecule expression within the microvasculature of human colorectal malignancies. *Clin Immunol Immunopathol* **72**: 129–136

Nguyen M, Strubel NA and Bischoff J (1993) A role for sialyl Lewis-X/A glycoconjugates in capillary morphogenesis. *Nature* **365**: 267–269

Nguyen M, Watanabe H, Budson AE, Richie JP, Hayes DF and Folkman J (1994) Elevated levels of an angiogenic peptide, basic fibroblast growth factor, in the urine of patients with a wide spectrum of cancers. *J Natl Cancer Inst* **86**: 356–361

Nicosia RF and Bonanno E (1991) Inhibition of angiogenesis in vitro by Arg-Gly-Asp-containing synthetic peptide. *Am J Pathol* **138**: 829–833

Orlidge A and D'Amore PA (1987) Inhibition of capillary endothelial cell growth by pericytes and smooth muscle cells. *J Cell Biol* **105**: 1455–1462

Paku S and Paweletz N (1991) First steps of tumor-related angiogenesis. *Lab Invest* **65**: 334–346

Paweletz N and Knierim M (1989) Tumor-related angiogenesis. *Crit Rev Oncol Hematol* **9**: 197–242

Pezella F, Dibacco A, Andreola S, Nicholson AG, Pastorino U and Harris AL (1996) Angiogenesis in primary lung-cancer and lung secondaries. *Eur J Cancer* **32A**: 2494–2500

Sakamoto N, Iwahana M, Tanaka NG and Osada Y (1991) Inhibition of angiogenesis and tumor growth by a synthetic laminin peptide, CDPGYIGSR-NH2. *Cancer Res* **51**: 903–906

Schnaper H, McGowan K, Kim-Schulze S and Cid M (1996) Oestrogen and endothelial cell angiogenic activity. *Clin Exp Pharmacol Physiol* **23**: 247–250

Sholley M, Ferguson G, Hr S, Montour J and Wilson J (1984) Mechanisms of neovascularization; vascular sprouting can occur without proliferation of endothelial cells. *Lab Invest* **51**: 624–634

Skinner SA, Frydman GM and O'Brien PE (1995) Microvascular structure of benign and malignant tumours of the colon in humans. *Dig Dis Sci* **40**: 373–384

Toi M, Hoshina S, Takayanagi T and Tominaga T (1994) Association of vascular endothelial growth factor expression with tumor angiogenesis and with early relapse in primary breast cancer. *Jpn J Cancer Res* **85**: 1045–1049

Van Hoef MEHM, Knox WF, Dhesi SS, Howell A and Schor AM (1993) Assessment of tumour vascularity as a prognostic factor in lymph node negative invasive breast cancer. *Eur J Cancer* **29**: 1141–1145

Vartanian RK and Weidner N (1994) Correlation of intratumoral endothelial-cell proliferation with microvessel density (tumor angiogenesis) and tumor-cell proliferation in breast-carcinoma. *Am J Pathol* **144**: 1188–1194

Warren B (1997) The vascular morphology of tumors. In: *Tumor Blood Circulation*, Peterson H (ed), pp. 1–47. CRC Press: Boca Raton

Weidner N, Semple JP, Welch WR and Folkman J (1991) Tumour angiogenesis and metastasis-correlation in invasive breast carcinoma. *N Engl J Med* **324**: 1–8

Weidner N, Folkman J, Pozza F, Bevilacqua P, Allred EN, Moore DH, Meli S and Gasparini G (1992) Tumor angiogenesis: a new significant and independent prognostic indicator in early-stage breast carcinoma. *J Natl Cancer Inst* **84**: 1875–1887

*© 2000 Cancer Research Campaign*
